# Tumor-Targeted Responsive Nanoparticle-Based Systems for Magnetic Resonance Imaging and Therapy

**DOI:** 10.1007/s11095-014-1436-x

**Published:** 2014-06-13

**Authors:** Ronak Savla, Olga B. Garbuzenko, Suzie Chen, Lorna Rodriguez-Rodriguez, Tamara Minko

**Affiliations:** 1 Department of Pharmaceutics, Ernest Mario School of Pharmacy, Rutgers, The State University of New Jersey, Piscataway, New Jersey 08854 USA; 2 Department of Chemical Biology, Ernest Mario School of Pharmacy Rutgers, The State University of New Jersey, Piscataway, New Jersey 08854 USA; 3 Rutgers Cancer Institute of New Jersey, New Brunswick, New Jersey 08903 USA; 4 Environmental and Occupational Health Sciences Institute, Piscataway, New Jersey 08854 USA; 5 Department of Obstetrics and Gynecology, Robert Wood Johnson Medical School, Rutgers, The State University of New Jersey, Piscataway, New Jersey 08901 USA; 6 Department of Pharmaceutics, Ernest Mario School of Pharmacy Rutgers, The State University of New Jersey, 160 Frelinghuysen Road, Piscataway, New Jersey 08854-8020 USA

**Keywords:** LHRH targeting peptide, manganese oxide nanoparticles, MMP2 cleavable peptide, nanostructured lipid carriers, vemurafenib

## Abstract

**Purpose:**

Design and synthesis of a tumor responsive nanoparticle-based system for imaging and treatment of various cancers.

**Methods:**

Manganese oxide nanoparticles (Mn_3_O_4_ NPs) were synthesized and modified with LHRH targeting peptide or anti-melanoma antibodies (cancer targeting moieties) and a MMP2 cleavable peptide (a possible chemotactic factor). Nanostructured lipid carriers (NLCs) were used to entrap the BRAF inhibitor, vemurafenib, and enhance cytotoxicity of the drug. Size distribution, stability, drug entrapment, cytotoxicity and genotoxicity of synthesized nanoparticles were studied *in vitro*. Enhancement of MRI signal by nanoparticles and their body distribution were examined *in vivo* on mouse models of melanoma, ovarian and lung cancers.

**Results:**

Uniform, stable cancer-targeted nanoparticles (PEGylated water-soluble Mn_3_O_4_ NPs and NLCs) were synthesized. No signs of cyto-,genotoxicity and DNA damage were detected for nanoparticles that do not contain an anticancer drug. Entrapment of vemurafenib into nanoparticles significantly enhanced drug toxicity in cancer cells with targeted V600E mutation. The developed nanoparticles containing LHRH and MMP2 peptides showed preferential accumulation in primary and metastatic tumors increasing the MRI signal in mice with melanoma, lung and ovarian cancers.

**Conclusions:**

The proposed nanoparticle-based systems provide the foundation for building an integrated MRI diagnostic and therapeutic approach for various types of cancer.

## INTRODUCTION

Theragnostics is an emerging field in medical research that can potentially improve overall patient therapy and outcome by combining diagnostic and specific therapeutic properties (1). Numerous research groups use various nanotechnology approaches for developing theragnostic agents. Although nanoparticles allow for distinct advantages in the field of therapy of different diseases, a combination of both diagnostic and therapeutic modalities in a single nanoparticle may be associated with certain difficulties. While nanoparticle properties can be fine-tuned in regards to the size, surface groups, and surface charge, synthesis of such a particle will be challenging due to the sheer number of modification steps involved. In addition, a single particle with both diagnostic and therapeutic capabilities may result in unnecessary treatment of false-positive tissues. Therefore, it may be ideal to develop a separated, but integrated multi-particle system for theragnostics.

Medical imaging procedures such as computed tomography (CT), X-ray, positron emission tomography (PET) and magnetic resonance imaging (MRI) are very commonly utilized in the clinic for the diagnosis and prognosis of various diseases. Designing nanoparticle contrast agents for these imaging modalities is an attractive and important goal. Each imaging modality has its strengths and limitations and the use of several type of imaging agents in combination may gain additional benefits in terms of revealing hidden details and improving the reliability of a diagnosis. MR imaging often represents the preferred modality for its cost efficacy and the non-invasive and non-ionizing nature (2). In addition, MRI provides anatomical images at high resolutions (3) thus making it an attractive imaging approach for the detection of small tumors and metastases.

Resolution of target structure or tissue can be enhanced by the use of MRI contrast agents, which are sub-divided into either T1-positive (paramagnetic) or T2-negative (superparamagnetic) agents (4). Gadolinium chelates have been FDA approved for use as contrast agents for MRI. However, possible induction of nephrogenic systemic fibrosis is a concern with these agents. In addition, the small size of the chelate imaging agents results in short circulation times (5). This may result in insufficient concentrations accumulated at spots of interests. It was concluded that a circulation half-life of 2 to 6 h would allow for adequate accumulation at target sites and therefore increased contrast (6). Nanoparticles have been extensively investigated for the delivery of small molecules and nucleic acids and as well as for imaging purposes (7). In addition, many types of nanoparticles have been investigated as optical imaging probes. Although optical imaging works well for small animals, its utilization for larger animals and humans represent certain difficulties. In addition, optical approach is not ideal for imaging of deeper anatomical structures (8). Moreover, instruments for optical imaging have yet to be fully developed for humans. Consequently, MRI especially in combination with nanoparticle-based contrast agents represents the most valuable approach for the detection of primary tumors and metastases.

It is known that nanoparticles coated with polyethylene glycol (PEG) polymer chains (PEGylated) may achieve circulation times up to several days (6). With the customizable nature of properties of such nanoparticles, nanocarriers for contrast enhancing agents with an optimal circulation time can be designed for imaging. In addition, the surface of such long-circulating nanocarriers can be modified with targeting ligands to improve targeting of imaging moieties specifically to cells and tissues of interest, *e.g.* primary localized and spreading tumors as well as metastases. Previously we have shown that many types of cancer cells overexpress luteinizing hormone releasing hormone (LHRH) receptors and LHRH peptide was successfully used in our laboratory as a targeting moiety for delivering drugs to tumors (9–17). Most imaging nanoparticles contain metal oxides and the cores of these particles are frequently synthesized at high temperatures and in inert atmospheres (18). Superparamagnetic iron oxide nanoparticles (SPIONs) are often employed as T2-negative contrast agents. However, the utilization of SPIONs as MRI contrast enhancers is hindered due to their negative contrast effect and tendency to include artifacts in the images (19). Recently, manganese oxide nanoparticles have begun to be investigated as positive MRI contrast agents (2,19–23). However, manganese-based contrast agents are not as well-studied as SPIONs.

Nanoparticle-based contrast agents are endowed with their imaging capabilities primarily due to the properties of their cores. Since the nanocrystal cores of such nanoparticles are not water soluble, a coating layer is required in order to provide for intravascular injection or oral delivery and transport of the nanoparticles in the systemic circulation. The most common coatings for manganese-based contrast agents are silica shells (3,4) and lipid-PEG molecules (22,24). In the form of coated nanoparticles, manganese atoms have a little contact with water and therefore bulk arrangement of atoms as in nanoparticles have low molar relaxivities and result in low positive contrast (20). When Mn^2+^ ions are released from the bulk nanocrystal structure inside cells after endocytosis (mainly under the action of low pH in endosomes), the ability of magnesium compounds to increase the relaxation rates of the surrounding water proton spins and therefore molar relativity may increase 10-fold.

Once the disease is diagnosed, it is important to deliver therapeutic agents to treat or cure the disease. Cytotoxic drugs are widely used for therapy of many types of cancers. Despite improvements in drug design and chemotherapy combinations, several challenges still limit the efficiency of the treatment. Conventional small molecule chemotherapeutic drugs lack specificity, possess high toxicity, and frequently induce drug resistance. Improvements in genetic analysis of cancers and computation have led to design of more selective and more potent drugs. Yet, the challenges faced by conventional chemotherapeutics have not been completely overcome, by only mitigated in part.

Nanoparticle-based delivery of chemotherapeutics has become a widely spreading area of research. Polymers, dendrimers, liposomes, emulsions, and inorganic nanoparticles have been extensively studied for the delivery of small molecules (13,14,25–31). More recently, solid lipid nanoparticles (SLNs) and nanostructured lipid carriers (NLCs) have begun to be increasingly investigated to overcome problems associated with other nanoparticle carriers (15,32–34). The most of novel and potent chemotherapeutic drugs tend to have very poor water solubility; therefore, entrapment in a lipid-based carrier may substantially improve their bioavailability and enhance the treatment efficacy. Although emulsions and liposomes are typically synthesized with well-studied excipients and can be produced on large scales, they have a limited ability to protect and modulate release of loaded drugs (35). Whereas SLNs are colloids consisting of solid lipids, NLCs contain liquid lipid entrapped within the voids of a spatially different solid lipid matrix (36). The liquid lipid appears not to affect the crystal structure of the solid lipid (37). Lipophilic drugs are solubilized in the liquid lipid and thus entrapped within the solid lipid matrix. Therefore, the loading capacity and effective drug release of NLCs is better when compared with SLNs (34,37).

In the present study, we have designed and tested biocompatible manganese oxide nanocrystals, which are capable of enhancing diagnosis and imaging of melanoma, ovarian and lung cancers using monoclonal antibodies or peptide cancer targeting moieties. We also demonstrated the ability to specifically target primary and metastatic tumors and diagnose them with efficient positive contrast ability. In addition, a cleavable peptide was conjugated onto the surface of the developed nanoparticles to act as a potential chemotactic messenger. Once deposited at tumor sites, matrix metalloproteinases can cleave a fragment off of the attached peptide. The detached peptide can diffuse away from the tumor site and into the systemic circulation. A concentration gradient of cleaved peptide fragments can be established, higher in proximity to the tumor site and lower farther away from tumor. Therapeutic nanoparticles modified with avidin can bind to and potentially be recruited to the tumor site. This presents a novel paradigm in targeted delivery whereby the target is not a cell-surface receptor on cancer cells, but rather a messenger molecule. A chemotherapeutic agent, vemurafenib, was furthermore entrapped in NLCs in order to effectively reach sites of melanoma tumors and metastases.

## MATERIALS AND METHODS

### Materials

Manganese (II) chloride, oleic acid, N-hydroxysuccimide (NHS), 3,3′,5,5′-tetramethylbenzidine (TMB), oleylamine, phosphoric acid (>85% wt.), calcium chloride, ethylenediaminetetraacetic acid (anhydrous, Sigma grade, approx. 99% titration, EDTA), 4-(2-hydroxyethyl)-1-piperazineethanesulfonic acid (HEPES), sodium chloride, hydrochloric acid (molecular biology grade, 36.5–38.0%), hydroxylamine hydrochloride (ReagentPlus®, 99%), Tween® 80, and S-acetylthioglycolic acid N-hydroxysuccinimide ester (SATA, ≥ 95% TLC) were purchased from Sigma-Aldrich (St. Louis, MO). Chloroform (HPLC grade), dimethyl sulfoxide (DMSO), reagent alcohol, xylenes, sodium dodecyl sulfate (SDS), N,N-dimethylformamide (DMF), sodium hydroxide (10 N solution, 30% *w/w*), 1× phosphate buffered saline (PBS) were purchased from Fisher Scientific (Fair Lawn, NJ). Pierce® Avidin, 1-ethyl-3-[3-dimethylaminopropyl]carbodiimide hydrochloride (EDC) were purchased from Thermo Scientific (Rockford, IL). SYBR® Gold nucleic acid gel stain was purchased from Life Technologies (Carlsbad, CA). Isopropyl myristate (96%) and 1-octadecene were purchased from Acros Organics. Cy5.5 Mono NHS-Ester was supplied from GE Healthcare Life Sciences (Piscataway, NJ). 1,2-distearoyl-*sn*-glycero-3-phosphoethanolamine-N-[Maleimide(Polyethylene Glycol) 2000] ammonium salt (DSPE-PEG-MAL), 1,2-distearoyl-*sn*-glycero-3-phosphoethanolamine-N-[Amino(Polyethylene Glycol) 2000] ammonium salt (DSPE-PEG-NH_2_), soy L-α-phosphatidylcholine (soy PC), and 1,2-dioleolyl-3-trimethylammonium-propane chloride salt (DOTAP) were purchased from Avanti Polar Lipids (Alabaster, AL). Anti-Melanoma antibody ab733 was purchased from Abcam (Cambridge, MA). Precirol® ATO 5 was a kind gift from Gattefosse (Paramus, NJ). Vemurafenib was purchased from Selleckchem (Houston, TX). Custom luteinizing hormone releasing hormone (LHRH) and cleavable by matrix metalloproteinase 2 (MMP2) peptides were manufactured by American Peptide Company (Sunnyvale, CA) according to our design. The LHRH targeting peptide had the amino acid sequence of Gln-His-Trp-Ser-Tyr-D-Lys(D-Cys)-Leu-Arg-Pro-NHEt. A custom MMP2 peptide contained the sequence of Biotin-Gly-Pro-Leu-Gly-Ile-Ala-Gly-Gln. Human active, recombinant MMP2 enzyme (MW 66 kDa) was purchased from EMD Millipore (Darmstadt, Germany).

### Synthesis of Mn_3_O_4_ Hydrophobic Cores

Manganese oxide (Mn_3_O_4_) hydrophobic cores were synthesized according to the published protocol (24). Briefly, one mmol of manganese (II) chloride, two mmol of oleic acid, and ten mmol of oleylamine were dissolved in 15 mL of xylenes at atmospheric conditions. The solution was heated and once it reached 90ºC, 3 mL of deionized water was added. The reaction was allowed to age at 90ºC for 3 h. The hydrophobic cores were precipitated with the addition of reagent alcohol and isolated by centrifugation. The dry cores were waxy and weighed before being dispersed in chloroform.

### Synthesis of Water Soluble Mn_3_O_4_ Nanoparticles (NH_2_/MAL Mn_3_O_4_ NPs)

Hydrophobic Mn_3_O_4_ cores (10 mg), 20 mg of DSPE-PEG-NH_2_ and 5 mg of DSPE-PEG-MAL were mixed in 2 mL chloroform. After proper mixing, the reaction mixture was heated to 60ºC and 8 mL of DMSO was slowly added. The reaction was stopped after all of the chloroform had evaporated. The used solvent exchange method is more appropriate when compared with other coating methods such as thin film hydration because the resulting hydrophilic particles are more uniform and stable.

### Conjugation of MMP2 Cleavable Peptide (MMP2/MAL Mn_3_O_4_ NPs)

A custom peptide (biotin-Gly-Pro-Leu-Gly-Ile-Ala-Gly-Gln) that is cleaved specifically by MMP2 enzyme (38) was attached to NH_2_/MAL Mn_3_O_4_ NPs using amide bond. The carboxy terminal of the MMP2 cleavable peptide would react with the surface free amines on the NH_2_/MAL Mn_3_O_4_ NPs. The free carboxyl on the MMP2 cleavable peptide was activated using EDC and NHS cross linkers. After 15 min, NH_2_/MAL Mn_3_O_4_ NPs were added. The reaction was allowed to continue for 24 h. The resultant conjugate was dialyzed against deionized water.

For *in vivo* biodistribution studies and peptide cleavage study, the MMP2 cleavable peptide without biotin (Gly-Pro-Leu-Gly-Ile-Ala-Gly-Gln) was reacted with Cy5.5-NHS ester. After 24 h of allowing for conjugation, the resulting Cy5.5-MMP2 was conjugated onto NH_2_/MAL Mn_3_O_4_ NPs as described above to yield Cy5.5-MMP2/MAL Mn_3_O_4_ NPs.

### Attachment of LHRH Targeting Moiety (MMP2/LHRH Mn_3_O_4_ NPs)

Previous studies have shown the ability of LHRH peptide (Gln-His-Trp-Ser-Tyr-DLys(DCys)-Leu-Arg-Pro-NHEt) to target various delivery systems specifically to different cancer cells (10–12). After attaching the MMP2 cleavable peptide, a thioether linkage was synthesized using the maleimide moiety on the MMP2/MAL Mn_3_O_4_ NP and the cysteine residue on LHRH peptide. In 100 mM phosphate buffer, 10 mg of custom LHRH peptide was reacted with previously synthesized MMP2/MAL Mn_3_O_4_ NPs. The reaction was performed at 4ºC overnight. The resultant conjugate was dialyzed against deionized water. The presence and amount of attached peptide was confirmed and measured using the Pierce® Bicinchoninic Acid (BCA) Protein Assay Kit (Thermo Scientific, Rockford, IL).

### Attachment of Anti-Melanoma Antibody (MMP2/ab733 Mn_3_O_4_ NPs)

The antibody modification and attachment protocol was adapted from Na *et al.* (22). An anti-melanoma antibody cocktail was purchased from Abcam (Cambridge, MA). The mixture is able to target neuraminidase-sensitive side chain in immature melanosomes (HMB45 antibody), melanoma antigen recognized by T cells (MART-1, DT101 and BC199 antibodies), and tyrosinase (T311 antibody). Briefly, ab733 Anti-Melanoma antibodies in 0.5 mL PBS was reacted with 60 μL of SATA in DMSO (1.5 mg/mL) for 30 min. Afterwards, 120 μL hydroxylamine (0.5 M in PBS) was added and the reaction was allowed for 2 h at room temperature. The modified antibody was purified using Amicon® centrifugal device (MWCO 30 kDa, Millipore, Billerica, MA). The purified product was added to MMP2/MALMn_3_O_4_ NPs in 100 mM phosphate buffer. The reaction was performed at 4ºC overnight. The resultant conjugate was dialyzed against deionized water.

### Quantification of Manganese Content in Mn_3_O_4_ NP Formulations

Manganese concentration in the NP formulation was assessed using a colorimetric assay. The original assay, developed for photosynthetic membranes (39), was adapted for assessing the manganese content of NPs. Briefly, conjugates were digested using concentrated hydrochloric acid. After the particles were disintegrated, the solution was diluted with deionized water and concentrated sodium hydroxide solution was added to create a basic pH solution. 3,3′,5,5′-tetramethylbenzidine (TMB) dissolved in 0.1 M hydrochloric acid was added and a precipitate was formed. Addition of 5.3 M phosphoric acid dissolved the crystals. The absorbance of the solution was measured at 450 nm wavelength. The manganese concentration was calculated based on a calibration curve.

### MMP2 Gene Expression

RNA was isolated from ovarian cancer cell lines using RNeasy kit (Qiagen, Frederick, MA) following the manufacturer’s protocol. Ready-To-Go You-Prime First Strand Beads (Amersham Biosciences, Piscataway, NJ) was mixed with 1 μg of total RNA and 100 ng of random hexadeoxynucleotide primer (Amersham Biosciences, Piscataway, NJ) to synthesize cDNA. The reaction mixture was subjected to quantitative polymerase chain reaction (qPCR) using SYBR® Green Master Mix as a detection agent. Qiagen (Frederick, MA) software was used to calculate difference in MMP2 gene expression between the cell lines by first normalizing the MMP2 gene expression in each cell line with that of β-actin, a housekeeping gene.

### Digestion of MMP2-Cleavable Peptide

The protocol was adapted from a previous study (38). A small amount of Cy5.5-MMP2/LHRH Mn_3_O_4_ NPs and 1 mg/mL of human MMP2 enzyme was mixed in HEPES buffered saline. At selected time intervals, a small amount was removed and centrifuged using Amicon® centrifugal device (MWCO 30 kDa, Millipore, Billerica, MA). The retentate was re-dissolved in buffer and the fluorescence signal was measured for Cy5.5 (λ_ex_ = 670 nm, λ_em_ = 690 nm). Cy5.5 dye was conjugated to the terminal end of the MMP2 cleavable peptide. Therefore, when the peptide is cleaved between glycine and isoleucine, the portion of Cy5.5-Gly-Pro-Leu-Gly is no longer attached to the surface of the nanoparticles and thus can be removed by centrifugal filtration.

### Synthesis of Nanostructured Lipid Carriers (NLCs)

A previously published protocol from our group was adapted for this project (15). Briefly, a melted ultrasonic dispersion method was utilized to synthesize vemurafenib (Vem) containing NLCs. Vemurafenib dissolved in DMSO was mixed into a hot lipid phase, which consisted of 100 Precirol ATO 5 (Gattefossé USA, Paramus, NJ), 100 mg isopropyl myristate, and 5 mg soy PC. The aqueous phase was composed of 250 mg Tween® 80, 10 mg DSPE-PEG-NH_2_, and 25 mg DOTAP in 10 mL deionized water. Both phases were heated to 80ºC in a silicone oil bath. After 15 min, the lipid phase was added to the aqueous phase while homogenizing at 12,000 rpm for 5 min using a high-speed homogenizer (PRO Scientific Inc., Oxford, CT). The emulsion was subject to ultrasonication (Model 120 Sonic Dismembrator, Fisher Scientific, Fairlawn, NJ) for 5 min at 3 W. The resulting emulsion was cooled to 4ºC in ice while being magnetically stirred. The NLC emulsion was dialyzed against deionized water using a membrane (MWCO 15 kDa) at 4ºC overnight. The dialyzed emulsion was stored at 4ºC. Empty NLCs were prepared in the same manner with equal volume of DMSO instead of vemurafenib.

### Vemurafenib HPLC Calibration Curve

Previously, a published protocol was modified for measuring vemurafenib concentration by HPLC (40). The chromatography system consisted of a Waters 1,525 with a Binary pump, Waters 2,487 Dual wavelength absorbance detector, Waters 717 plus autosampler, and Breeze workstation (Waters Corp, Milford, USA). The stationary phase consisted of a C8 Xterra® MS (250 mm × 4.6 mm, 5 μm; Waters Corp, Milford, USA) column with a guard column and a mixture of 100 mM glycine buffer (pH 9.00) and acetonitrile (45:55, *v/v*) was used for the mobile phase. Each sample was subjected to a 15 min run at a flow rate of 0.9 mL/min at 30ºC. The UV detector was used to detect vemurafenib at a wavelength of 249 nm.

### Vemurafenib Entrapment Efficiency and Loading in NLCs

Due to the high hydrophobicity of vemurafenib, the concentration of free dissolved in the water phase drug is negligible. In order to measure the concentration of bound drug, the NLC-vemurafenib conjugates were disrupted by mixing with 4× volume of methanol. Then, the mixture was sonicated for 10 min and centrifuged at 3,500 rpm for 15 min. The supernatant was collected and analyzed using described above HPLC protocol.

### NLC Drug Entrapment Stability

The entrapment stability of vemurafenib in NLC formulation was studied using dialysis. A small volume of Vem-NLC dispersion was loaded in a dialysis membrane (Spectra/Por® Biotech RC dialysis membrane, MWCO 15 kDa, Spectrum Laboratories Inc., Houston, TX) and placed in phosphate buffered saline containing 15% ethanol and stirred with a magnetic stirrer at 37ºC. At pre-determined time intervals, a small amount of Vem-NLC dispersion was removed from the membrane as well as the medium. The Vem-NLC sample was disrupted as above and both samples (Vem-NLC and medium) were run on HPLC to assess vemurafenib concentration.

### Size Distribution, Cytotoxicity and DNA Damage

The size distributions of MMP2/LHRH Mn_3_O_4_ NPs and Vem-NLC formulations were measured using Malvern ZetaSizer NanoSeries (Malvern Instruments, Malvern, UK) according to the manufacturer’s recommendations. Measurements were performed at 25ºC. The sizes were measured five times, and average values were calculated. The cytotoxicity of MMP2/LHRH Mn_3_O_4_ NPs and empty NLCs were assessed using a modified MTT (3-(4,5-dimethylthiazol-2-yl)-2,5-diphenyltetrazolium bromide) assay as previously described (41). Briefly, CHO-K1 cells (a model for normal cells) were plated in a 96 well plate at a density of 10,000 cells/well. Twenty-four hours after seeding, different concentrations MMP2/LHRH Mn_3_O_4_ NP conjugates were added to the cells. Control cells were treated with an equal volume of fresh medium. The cells were treated for 24 h. The liquid was removed and replaced with fresh medium and MTT solution. After further 3 h incubation, a solubilizing solution was added and the plates were stored overnight. The absorbance intensity was measured the following day. The cytotoxicity of drug-containing formulations was measured using metastatic melanoma cell line, Colo 829, which expressed the V600E mutation. The following formulations were assessed: free non-conjugated vemurafenib (Vem) and vemurafenib entrapped in NLCs (Vem-NLC).

The CometAssay® HT kit (Trevigen, Gaithersburg, MD) was used as a sensitive genotoxicity test for the detection of DNA damage induced by MMP2/LHRH Mn_3_O_4_ NPs and NLCs. Approximately 300,000 CHO-K1 cells were plated in a 25 cm^2^ cell culture flasks and incubated for 24 h prior to treatment. Cell culture medium was replaced with either fresh medium (negative control), medium containing 100 μM of hydrogen peroxide (positive control), non-cytotoxic concentration of MMP2/LHRH Mn_3_O_4_ NP (7.1 mg/l Mn^2+^) or empty NLCs (80 mg/l lipid concentration) for 24 h. After the treatment, the cells were pelleted and suspended in ice-cold PBS buffer at a concentration of 1 × 10^5^ cells/ml. The cell suspension was mixed with molten COMET Agarose at a ratio of 1:10 (*v/v*). A small aliquot was pipetted onto the surface of a COMET slide. After solidification, the slides were immersed in lysis solution at 4ºC for 30 min and then in alkaline unwinding solution for 60 min at room temperature. The slides were placed in a horizontal electrophoresis apparatus containing enough 1× TBE buffer to cover the samples. Minimal voltage was applied for 45 min. Afterwards, the slides were rinsed with deionized water and 70% ethanol. Samples were air dried to bring all cells into a single plane. DNA was stained with SYBR Gold (Life Technologies, Carlsbad, CA) for 30 min. The samples were rinsed with deionized water, dried, and viewed using a fluorescence microscope. DNA damage was assessed by calculating the percent of DNA in the comet tail using CometScore^™^ (TriTek Corp., Sumerduck, VA) software.

### Cancer Cells

Human lung (A549), ovarian (A2780), melanoma (COLO 829) cancer cells and Chinese hamster ovary cells (CHO-K1) were purchased from ATCC (Manassas, VA). A549 cells expressing luciferase enzyme were purchased from Xenogen Bioscience (Cranbury, NJ). The ascitic fluid with cancer cells was obtained from the peritoneum area of the patients with ovarian cancer provided by the Cancer Institute of New Jersey. The samples were centrifuged for 20 min at 2,000 g; the supernatant was discarded and cell pellets were consequently re-suspended. The re-suspended cells were cultured in RPMI 1,640 media with L-glutamine (Lonza, Walkersvile, MD) supplemented with 15% fetal bovine serum (Invitrogen, Carlsbad, CA), 2.5 μg/ml insulin (Sigma, St. Louis, MO) and 1.2 mL/100 mL penicillin-streptomycin (Gibco, Grand Island, NY). A549, A2780, and COLO 829 cells were grown in RPMI 1,640 medium (Lonza, Walkersville, MD) supplemented with 10% fetal bovine serum (Life Technologies, Carlsbad, CA) and 1.2 mL/100 mL penicillin-streptomycin (Life Technologies, Carlsbad, CA). CHO-K1 cells were grown in F12-K medium supplemented with 10% fetal bovine serum (Life Technologies, Carlsbad, CA). All cells were grown in a humidified atmosphere of 5% CO_2_ (*v/v*) at 37°C. All experiments were performed on the cells in the exponential growth phase.

### Animal Cancer Models

The current research adhered to the “Principles of Laboratory Animal Care” of NIH and was carried out according to the protocols approved by the Institutional Animal Care and Use Committee (IACUC). Nude mice were inoculated with human lung (A549) and ovarian (A2780) cancer cells or cells isolated from malignant ascites obtained from patients with ovarian carcinoma. All cancer cells were transfected with luciferase. Orthotopic lung cancer mouse model was created by intratracheal instillation of A549 lung cancer cells as previously described (15,42,43). Both orthotopic (intraperitoneal) and xenograft (subcutaneous) ovarian cancer models were created using A2780 or malignant ascites cells as previously described (9,16). Luciferase transfected cancer cells were visualized in live anesthetized animals using *in-vivo* bioluminescence IVIS system (Xenogen, Alameda, CA). Luciferin (150 mg/kg) was intraperitoneally administered 10–15 min before imaging. According to the IACUC policy, the size of subcutaneous tumor was measured daily using a caliper and the volume was calculated as d^2^ × D/2 where d and D are the shortest and longest diameters (in millimeters) of the tumor, respectively. Once the tumor volume reached 200–300 mm^3^, MMP2/LHRH Mn_3_O_4_ nanoparticles were injected intraperitoneally in the volume of 0.1 μL into the mice. For melanoma imaging studies, metabotropic glutamate receptor 1 (grm-1) transgenic mouse model was developed as previously described (13,44,45). The hallmark of the grm-1 transgenic mice is the spontaneous development of pigmented cutaneous melanomas. The melanoma development simulates that seen in humans where cancer first develops on the ears, then eyelids and then perianal region.

### *In vivo* Optical and Magnetic Resonance Imaging

Mice were anesthetized with isoflurane (4% for induction of anesthesia and 1–2% for maintenance) using XGI-8 Gas Anesthesia System (Xenogen, Alameda, CA) for all imaging procedures. MRI was performed using a 1 T M2™ whole body scanner (Aspect Imaging, Shoham, Israel). Images (repetition time 2,607 ms, echo time 44 ms) were recorded in Fast Spin Echo sequence. At a spatial resolution of 312 μm, the tumors were coronal imaged in a single section through the mouse body using an image matrix of 256 × 256, a field of view of 80 mm^2^, and 4 excitation. MR signal was calculated using Vivoquant 1.21 software (Invicro, Boston, MA). For MRI studies, mice were treated with 2 mg Mn^2+^ per kg body mass administered intraperitoneally (2) either in a free form or as the nanoparticle formulation.

### Statistical Analysis

Data were analyzed using descriptive statistics and single-factor ANOVA, and are presented as a mean ± SD from five to ten independent measurements. Ten animals were used in each experimental group. The power analysis (alpha = 0.05; power = 80%) based on the preliminary results showed that ten mice per group are needed for statistical analysis to determine a 15% difference between the groups. We analyzed data sets for significance with Student’s *t* test and considered *P* values of less than 0.05 as statistically significant.

## RESULTS

### Synthesis and Characterization of Water Soluble Manganese Oxide Nanoparticles

Uniform hydrophobic Mn_3_O_4_ were synthesized at a low temperature and normal atmospheric conditions. The reaction yielded an average of approximately 150 mg of these hydrophobic cores. The reaction time allowed for control of crystal growth. Longer aging time led to synthesis of a larger nanocrystal. The three hour aging time in this reaction yielded cores with an average diameter of 6.17 nm. Previous investigation assessed the X-ray diffraction pattern of the nanocrystal synthesized using this procedure and identified the crystal as having a tetragonal (hausmannite) structure (24).

These manganese oxide cores are very hydrophobic and must be coated for clinical use. In addition to imparting water solubility, the polymer coating protects the particles from aggregation, limits hydrophobic interactions with cells especially immune cells, helps to improve pharmacokinetics and allows for further surface modification (46,47). Using a dual solvent exchange method, the hydrophobic cores were coated with lipid-PEG molecules to make them water soluble and allow for further modification.

**Fig. 1:**
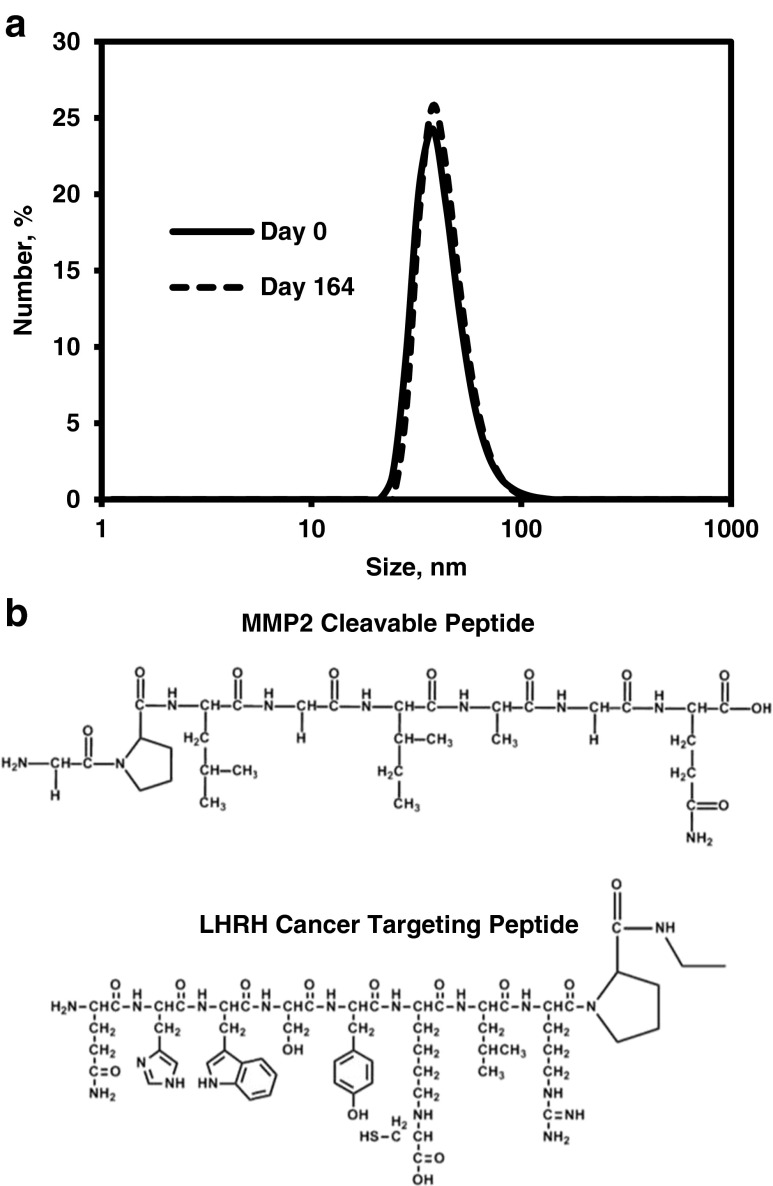
(**a**) Typical distribution of the size of PEGylated Mn_3_O_4_ nanoparticles before and after the storage (164 days). (**b**) Chemical structures of MMP2 cleavable and LHRH cancer targeting peptides.

When the core was coated with DSPE-PEG molecules, the average size increased to 42.17 ± 5.36 (mean ± SD) nm from 6.17 ± 0.76 (mean ± SD) nm (*P* < 0.05, Table I). The PEGylated Mn_3_O_4_ NPs displayed narrow size distribution and when stored at 4°C, they had excellent stability properties. At 164 days of storage, there were no significant changes in mean particle size or particle size distribution (Fig. 1a).

**Table I:** Size of Manganese Oxide Nanoparticle. Means ± SD are shown

Particles	Size,nm
Mn_3_O_4_ cores	6.17 ± 0.76
NH_2_/MAL Mn_3_O_4_ NPs	42.17 ± 5.36
MMP2/MAL Mn_3_O_4_ NPs	25.09 ± 2.99
MMP2/LHRH Mn_3_O_4_ NPs	26.68 ± 3.49

The resulting NH_2_/MAL Mn_3_O_4_ NPs were then modified first with a MMP2 cleavable peptide (Fig. 1b) and then with luteinizing hormone-release hormone (LHRH) receptor targeting peptide (Fig. 1c; please note a thioether linkage of maleimide moiety on DSPE-PEG molecule with a cysteine residue on the LHRH peptide) to enable active targeting to ovarian and lung cancers (12). Attachment of LHRH peptide was confirmed using BCA protein assay (Thermo Fisher Scientific Inc., Rockford, IL) according to manufacturer’s protocol. The results of the BCA Assay indicated that in average 1.52 ± 0.33 (mean ± SD) mg LHRH peptide was attached onto the surface of 10 mg of MMP2/LHRH Mn_3_O_4_ NPs. The concentration of MMP2 cleavable peptide was below the sensitivity level of the BCA Assay.

The MMP2/LHRH Mn_3_O_4_ NPs were digested with hydrochloric acid and purified to obtain a solution containing manganese ions. A protocol used for determination of manganese concentration in water and plants was modified for our purpose. Based on our standard curve (MnCl_2_ solution in deionized water), the Mn^2+^ concentration in the formulated MMP2/LHRH Mn_3_O_4_ NPs was 48.28 ± 7.59 (mean ± SD) mg/L. This concentration was used for manganese content in other experiments and was used for *in vivo* dosing (2 mg Mn^2+^ per kg mouse body weight).

**Fig. 2:**
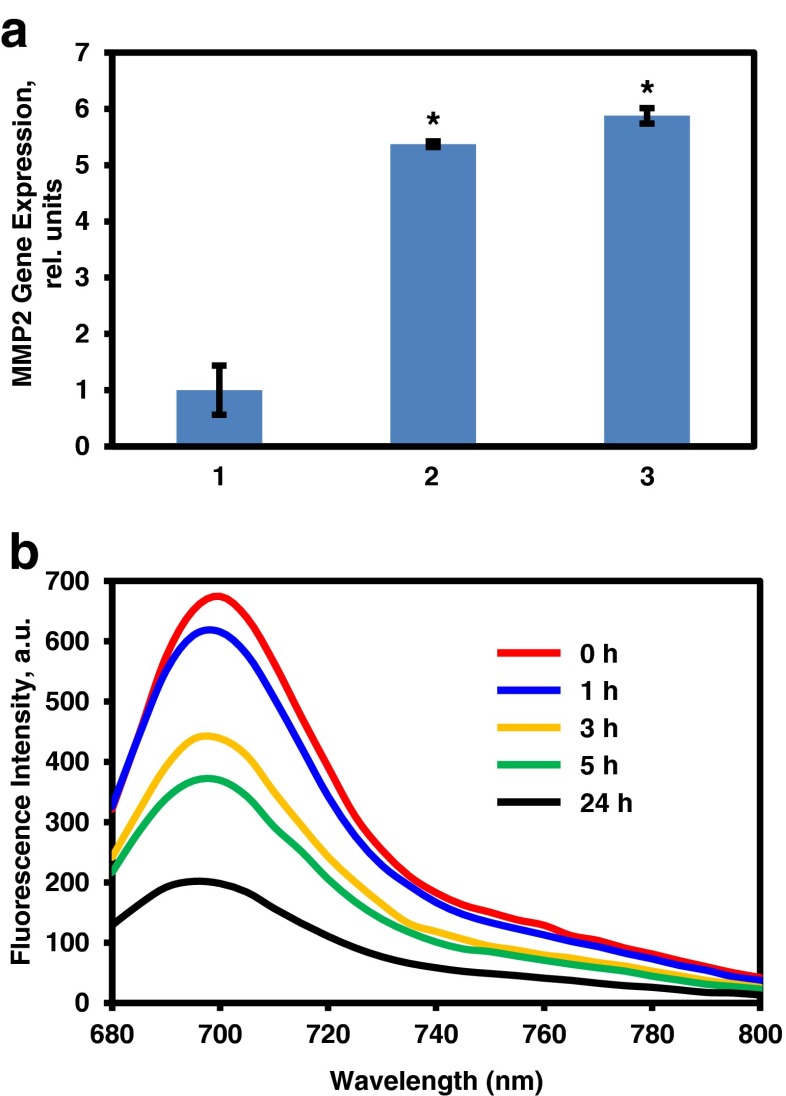
(**a**) Expression of MMP2 mRNA. The expression was measured by quantitative PCR in RNA isolated from: (1) A2780 human ovarian cancer cells; (2) cells from malignant ascites obtained from patients with ovarian carcinoma and grown *in vitro*; (3) tumor tissues from mice with xenografts of cancer cells from malignant ascites obtained from patients with ovarian carcinoma. Means ± SD are shown. *P < 0.05 when compared with A2780 human ovarian cancer cells. (**b**) Cleavage of MMP2 peptide after incubation with human MMP2 enzyme. Nanoparticles modified with cleavable MMP2 peptide were incubated with 1 mg/mL of human MMP2 enzyme in HEPES buffered saline. Representative fluorescence spectra are shown.

Matrix metalloproteinases are extracellular enzymes involved in a variety of functions including modification of the extracellular matrix. They are present is almost all cell types and are seen in increased quantity and activity in a variety of pathologies including cancers including ovarian and lung cancers and melanoma (48). Our results on two different ovarian cancer cell lines show that MMP2 is expressed nearly 5–6 folds higher in cells isolated from malignant ascites (*in vitro* cell culture and *ex vivo* tumor xenograft) obtained from patients with advanced ovarian carcinoma when compared with A2780 ovarian cancer cells (Fig. 2a).

The MMP2 peptide cleavage experiment showed that the fluorescence signal from the peptide labelled with Cy5.5 near infrared fluorescence dye decreased as the peptide was cleaved and the cleaved fragment removed (Fig. 2b). The cleavage between the glycine and isoleucine amino acids led to the dissociation of Cy5.5-Gly-Pro-Leu-Gly fragment, which was easily removed from the solution by centrifugal filtration. The data show that almost all peptide was cleaved from the NPs after 24 h.

### Synthesis and Characterization of Vem-NLCs

The average particle size of Vem-NLCs was 174.51 ± 31.76 (mean ± SD) nm. The entrapment of vemurafenib within the NLCs was quantified based on the equations below. Based on the HPLC calibration curve, our formulation was able to entrap 6.23 ± 1.09 (mean ± SD) mg of the 10 mg of vemurafenib used in the synthesis yielding an entrapment efficiency of 62.3%. The drug loading percentage was 3.04%.

$$ \begin{array}{c}\hfill EE\%=\left(\frac{ Wloaded}{ Wtotal}\right)\times 100\%\hfill \\ {}\hfill DL=\left(\frac{ Wloaded}{ Wlipid}\right)\times 100\%\hfill \end{array} $$

EE%-entrapment efficiency, DL%- drug loading, W_total_- weight of vemurafenib added during preparation, W_lipid_- weight of lipids used during preparation (Precirol ATO 5, Isopropyl myristate, Soy PC), W_loaded_- weight of vemurafenib entrapped in Vem-NLC.

One of the primary advantages of entrapping chemotherapeutics in nanoparticles is the reduction of free drug exposure in the plasma and off-target organs and tissues. An ideal nanocarrier would not release any drug in the blood circulation. Accumulation in tumor tissue by active targeting or enhanced permeation and retention (EPR) effect can allow for degradation of the nanocarrier and release of entrapped drug molecule. We examined by HPLC drug leakage and retention in the NPs in PBS buffer (pH = 7.4) within 72 h. The HPLC measurements did not reveal a measurable amount of vemurafenib in the dialysis buffer in these conditions. In contrast, the amount of vemurafenib still entrapped in NLCs did not change statistically significantly in the same conditions. There was no change in retention time of vemurafenib as detected by HPLC when assessing drug loading and particle stability. Therefore, we concluded that the NLCs can retain vemurafenib in plasma with normal pH for at least 72 h.

### Cytotoxicity and Genotoxicity of Nanoparticle Formulations

**Fig. 3:**
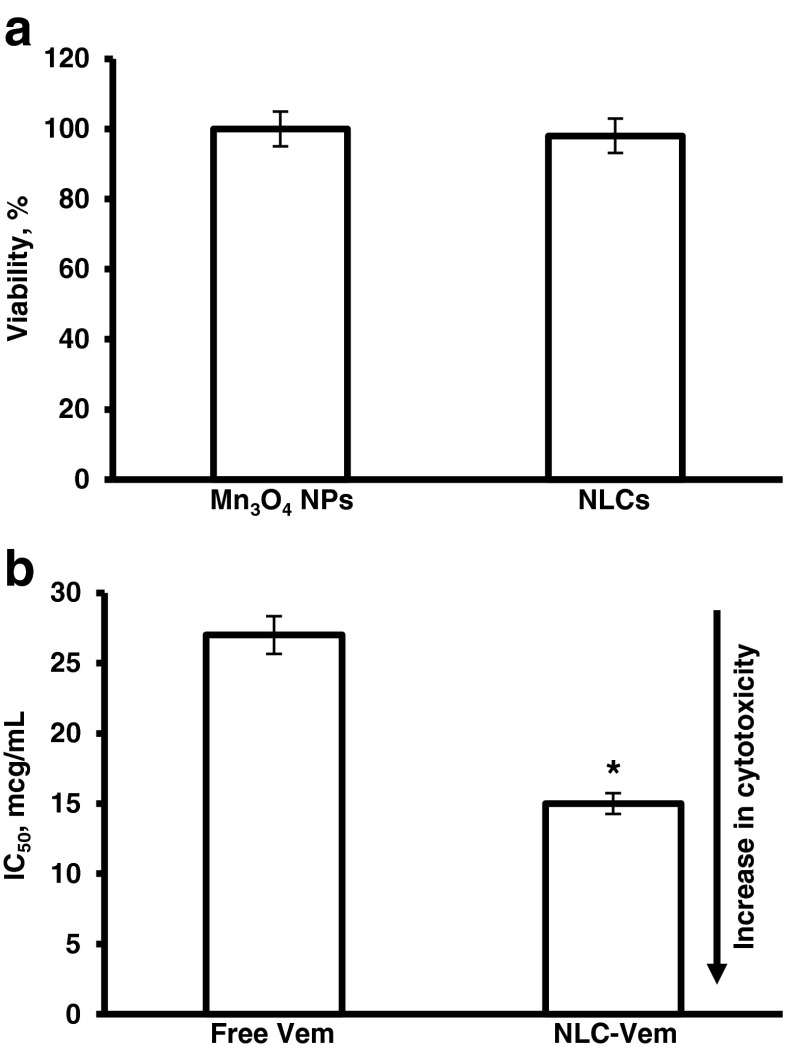
Cytotoxicity of nanoparticles (manganese oxide – Mn_3_O_4_ NPs and nanostructured lipid carriers – NLCs). (**a**) Cellular viability of cells incubated with nanoparticles that do not contain an anticancer drug. The cells were incubated 24 h with nanoparticles indicated. The concentrations of nanoparticles were 7.1 mg/L of Mn2+ and 80 mg/L of lipid for Mn^3^O^4^ and NLC, respectively. (**b**) Cytotoxicity of free non-conjugated vemurafenib (Vem) and NLC-encapsulated Vem. Colo 829 metastatic melanoma cells with the targeted V600E mutation were incubated with free and encapsulated in NLC-Vem. Means ± SD are shown. *P < 0.05 when compared with free vemurafenib.

**Figure 4:**
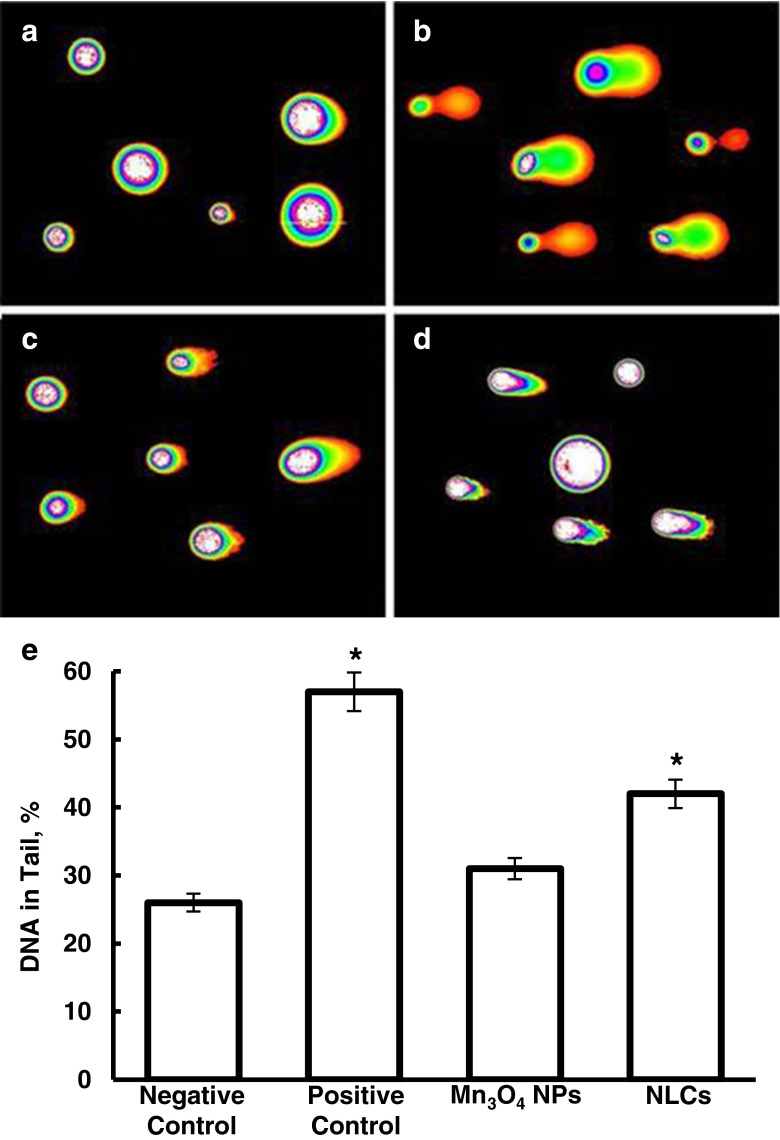
DNA damage potential of nanoparticles. (**a-d**) Representative fluorescence images of cells with DNA damage after treatment with (**a**) Negative control (fresh medium); (**b**) Positive control (hydrogen peroxide); (**c**) Mn_3_O_4_ NPs; (**d**) NLCs. (**e**) Quantitative analysis of DNA damage evaluated using a comet assay method. Means ± SD are shown. *P < 0.05 when compared with negative control.

Cytotoxicity of MMP2/LHRH Mn_3_O_4_ NPs and NLCs were assessed using CHO-K1 cells, which we used as a model for normal healthy tissue. The results show that MMP2/LHRH Mn_3_O_4_ NPs and empty NLCs are non-cytotoxic at concentrations used in other experiments (Fig. 3a). In addition, genotoxicity study demonstrated that empty MMP2/LHRH Mn_3_O_4_ NPs are not likely to induce DNA damage (Fig. 4). Negative (fresh medium) and positive (hydrogen peroxide) controls were used as references. The assay also revealed that empty NLCs do not cause appreciable DNA damage. Cytotoxicity of free vemurafenib and encapsulated vemurafenib was assessed using Colo829 melanoma cells, which contain the BRAF V600E mutation. It was found that encapsulation of vemurafenib within NLCs significantly increased its cytotoxicity (Fig. 3b).

### *In vivo* Animal Magnetic Resonance Imaging

**Fig. 5:**
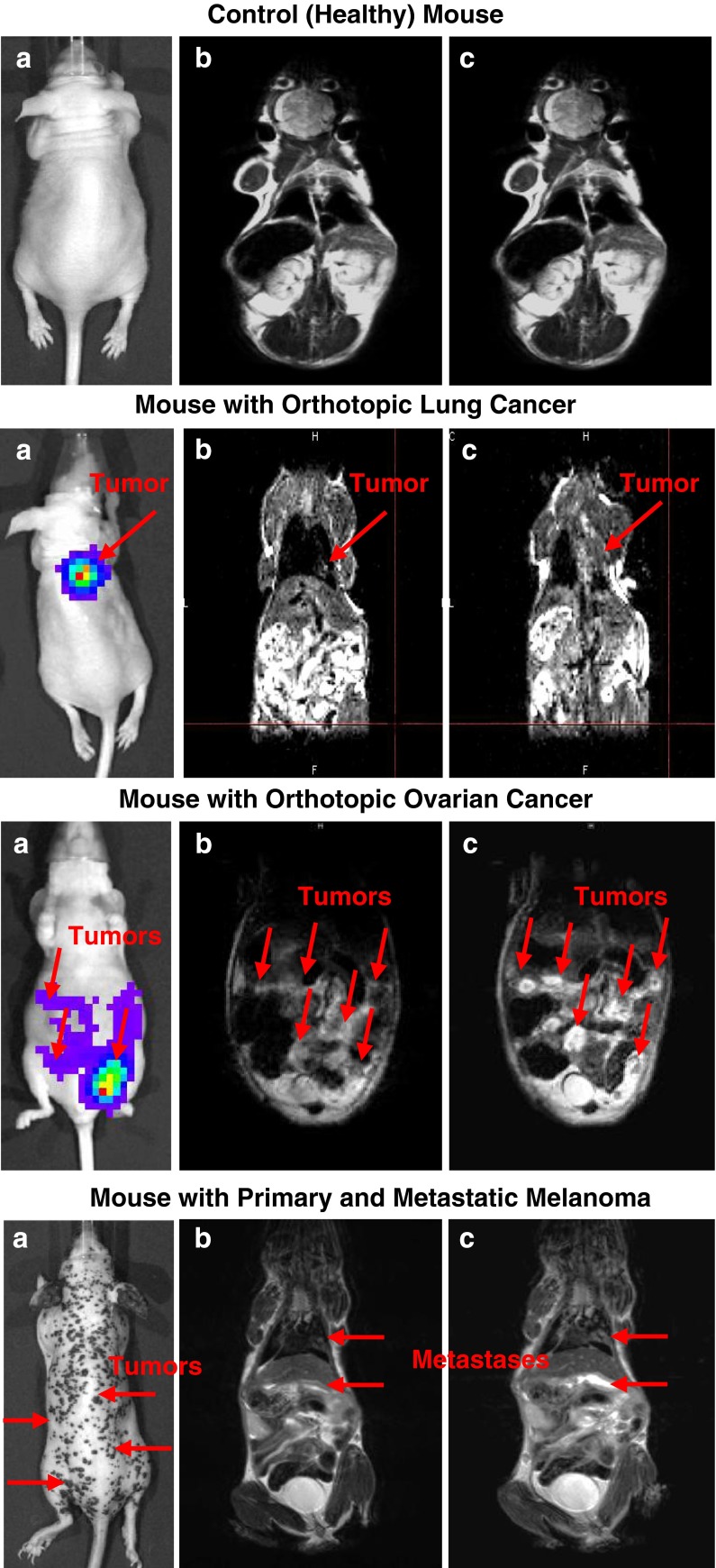
Enhancement in MRI sensitivity and specificity by cancer-targeted Mn_3_O_4_ nanoparticles. (**a**) Representative light or bioluminescence IVIS optical imaging. (**b-c**) Representative magnetic resonance imaging. (**b**) MRI without a contrast agent. (**c**) MRI after injection of biocompatible cancer-targeted Mn_3_O_4_ nanoparticles. Representative MR images are shown.

The Mn_3_O_4_ NPs were tested as imaging agents in various mouse models of cancer. The development of lung and intraperitoneal tumors *in vivo* was confirmed by the detection of bioluminescence of cancer cells using IVIS imaging system in anesthetized animals (Fig. 5a). The administration of MMP2/LHRH Mn_3_O_4_ NPs to the mice with orthotopic lung cancer model substantially improved the visualization of the cancer mass in the lungs. Prior to contrast administration, only a small mass is detected in the lower lobe of the right lung (Fig. 5b). After injection of NPs, cancer cells were clearly detected in the right lung (Fig. 5c). Consequently, the use of NPs as contrast agents increased signal in the entire lung and also brightened a potential metastatic mass on the periphery. In the intraperitoneal model of ovarian cancer, the administration of MMP2/LHRH Mn_3_O_4_ NPs not only increase signals in the cancer masses, but also allowed for outline of their shapes.

MMP2/ab733 Mn_3_O_4_ NPs were also used as the contrast agents for the detection of melanoma in transgenic mice. These mice spontaneously develop cutaneous melanoma lesions with frequent metastases. While the cutaneous lesions are easily identifiable, better procedures are required for detecting metastases. The use of NPs as contrast agents helped to detect internal metastases in the right lung and in the abdomen of the transgenic mice (Fig. 5c, lower panel).

**Fig. 6:**
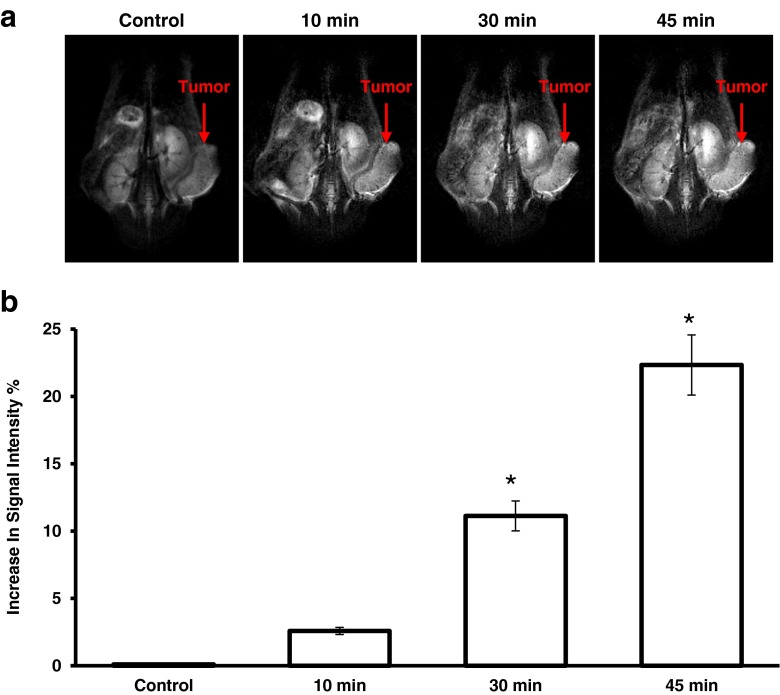
Distribution of cancer-targeted Mn_3_O_4_ nanoparticles and enhancement in MRI signal intensity. (**a**) Representative MR images of a mouse with subcutaneous xenografts of human ovarian cancer before (control) and 10, 30, and 45 min after injection of the cancer-targeted nanoparticles. (**b**) Quantitative analysis of MRI signal intensity. Means ± SD are shown. *P < 0.05 when compared with control.

In a subcutaneous ovarian cancer xenograft model, the tumor mass is visible on the right flank of the mouse (Fig. 6a). MMP2/LHRH Mn_3_O_4_ NPs were injected subcutaneously on the contralateral side of the mouse. The time lapse images show signal brightening at the site of the injection. More impressively, the images clearly show the diffusion of the tumor-targeted MMP2/LHRH Mn_3_O_4_ NPs from site of administration to the tumor and preferential accumulation in the primary tumor and metastases. Quantitative measurements revealed that 45 min post-injection, the MR signal in the tumor had increased by 22.3% (Fig. 6b).

## DISCUSSION

In the present investigation, the manganese cores were synthesized using manganese chloride, oleic acid, and oleylamine as precursors. As the manganese chloride dissolved, it gave the reaction solution a pinkish color. Later, this transitioned into an orange to brown color. The addition of deionized water turned the solution to a dark brown-black color. The color transition to a brown-black color indicates formation of nanoparticles (49). The reaction temperature plays a role in the core shape; high reaction temperatures lead to one dimensional rod formation whereas lower temperatures over a longer time yield spherical nanocrystals. In this method for crystallization, water acted as a reagent and the source for oxygen, oleylamine acted as a base to catalyze the nucleation of crystal, and the addition of oleic acid helped to create a spherical nanocrystal as compared to nanocrystal plates (24). The three hour aging time used for this study yielded particles with an average diameter around 6 nm. Increasing the aging time to 4 h resulted in the increase of average diameter of cores to approximately 18 nm. The smaller cores were used because of the potential for higher contrast resulting from the higher surface area to volume ratio for the smaller core compared to the larger core.

These hydrophobic cores cannot be used by themselves for therapy and must be converted into a water-soluble, biocompatible form. Thin film hydration is a commonly used technique to coat hydrophobic nanoparticle cores with lipid-PEG molecules to make water soluble nanoparticles. However, this process results in irreversible aggregation of nanoparticles, creation of empty micelles, and raises concerns of stability issues. Using a dual solvent exchange method (50), in which the hydrophobic cores are coated by changing the solvent from chloroform to dimethyl sulfoxide to water results in a higher surface coating density, more uniform distribution of nanoparticles, and more stable nanoparticles. Because the immiscibility of chloroform and water, an intermediary solvent, dimethyl sulfoxide, was used in the present study to transfer the core/lipid-PEG mixture into water as well as to induce coating of the cores through hydrophobic interactions. PEGylated Mn_3_O_4_ nanoparticles showed narrow size distribution and no change in mean particle size or size distribution after 164 days of storage (Fig. 1a).

The coating with lipid-PEG molecules endows free surface functional groups, amines and maleimides, for further modification. MMP2 cleavable peptide was conjugated to the free primary amines using a stable amide bond. LHRH cancer targeting peptide was also attached *via* the cysteine to the maleimide functional group forming a thioether bond. The attachment of LHRH peptide was confirmed using the BCA protein assay. The LHRH peptide developed a strong and clear absorbance signal in this assay because of the presence of aromatic structures on amino acid side chains in this peptide. According to the manufacturer, the color formation of the assay is principally due to the presence of certain amino acids (cysteine, tryptophan, and tyrosine). These three amino acids are present in the LHRH targeting peptide and are absent in the MMP2 cleavable peptide. This difference in the composition of these peptides explains a strong color development in the BCA assay for LHRH peptide and the absence of color formation in case of the MMP2 cleavable peptide.

It was interesting that when the water soluble nanoparticles were modified with MMP2 cleavable peptide, the average size decreased from 42.17 ± 5.36 (mean ± SD) nm to 25.09 ± 2.99 (mean ± SD) nm. This phenomenon could probably be attributed to a change in structure of the DSPE-PEG molecules (51). The PEG molecule on the surface of NH_2_/MAL Mn_3_O_4_ NPs likely maintains a “brush” configuration. The modification of NPs with the MMP2 cleavable peptide, changes its configuration to a “mushroom” configuration. Based on the data obtained, this configuration appears to be maintained after conjugation of LHRH targeting peptide. A nanoparticle size between 10 and 100 nm is typically sought for most intravenous applications. Nanoparticles larger than 200 nm are subject to rapid elimination by the reticuloendothelial system and those smaller than 10 nm can be efficiently cleared by the kidney (52). This size range also allows for passive tumor accumulation by the enhanced permeation and retention effect. The increase in size of MMP2/LHRH Mn_3_O_4_ NPs after coating with PEG showed that the used synthetic conditions led to the covering of nanoparticles with a substantial protective layer but did not result in a so-called “thick” coating. A thinner core coating allows for an easier passage of water to the core, liberation of Mn^2+^ ions, and quicker core digestion; all three processes will be amicable in increasing MR signal.

We hypothesized that the expression of MMP2 in the tumor microenvironment can be considered as a hallmark feature or cancers with high metastatic potential and, consequently, MMP2 cleavable peptide can be used to enhance the detection of metastases by MRI. In order to verify this hypothesis, we measured the MMP2 expression in established ovarian cancer cells and highly metastatic cancer cells isolated from malignant ascites obtained from patients with advanced ovarian carcinoma using quantitative PCR. The results of these measurements showed that highly metastatic cancer cells had a 5–6 - fold higher expression of MMP2 mRNA when compared with A2780 ovarian cancer cells. It is possible that the digestion of the peptide is correlated to the expression and activity of MMP2 enzyme. Consequently, the increase release of the biotin-fragment from the avidin-modified MMP2 peptide used in the present study after its cleavage in the tumor could potentially represent an attractive target for avidin-conjugated nanoparticles containing a therapeutic agent. We are planning to use such avidin-modified nanoparticles for targeted chemotherapy after the pretreatment with biotin modified MMP2 cleavable peptide on the surface of the Mn_3_O_4_ NPs. Once the biotin-Gly-Pro-Leu-Gly fragment is dissociated, it can act as a signal to recruit avidin-modified therapeutic drug containing nanoparticles to the tumor sites. This will provide a basis for the responsive theragnostic platform in which the therapeutic payload is preferentially delivered to tumors with high activity of MMP2 enzymes (*i.e.* containing highly metastatic cancer cells).

In the current study, two substantially different lipids were used for synthesis of NLC. Precirol® ATO 5 consists of glycerol distearate (type I) EP, glyceryl distearate NF, and glyceryl palmitostearate (USA FDA IIG). The combination of spatially different lipids results in an imperfect solid matrix. The addition of a specialized liquid lipid such as isopropyl myristate prevents the crystallization of the matrix to β forms caused an amorphous matrix (53). The combination of the two processes may yield a NLC with high drug loading and reduced drug expulsion. This appears to be evident from our results. Tween 80 and DSPE-PEG-NH_2_ were added to improve water solubility of the lipid carrier. The primary amine on the DSPE-PEG-NH_2_ can be used to add other functionalities in the future such as targeting ligands such as avidin. A study by Bocca *et al.* (54) showed that PEGylated or stealth solid lipid nanoparticles had reduced phagocytosis rate compared to non-stealth counterparts. The increased circulation time will increase the likelihood of the nanoparticles to be recruited to tumor sites.

There is a restriction on the use of gadolinium chelate MRI contrast agents because of the potential of nephrogenic systemic fibrosis . Several iron based contrast agents have been withdrawn from market due to safety concerns or discontinued by the manufacturer. GastroMARK® is the only available iron oxide contrast agent available and is approved for imaging of the gastrointestinal tract (55,56). However, performing solely a cytotoxicity assay does not adequately address safety concerns. Previously, we showed that even at safe levels, nanoparticles may impose negative effects on healthy tissues, *e.g.* induce genetic aberrations (57). The CometAssay® allows for the visualization of DNA damage caused by treatment with nanoparticles. After cells are treated with the nanoparticles, any DNA damage resulting in fragmentation appears as tails after electrophoresis. Long tails and more intense SYBR Gold® staining in the tail are indications of DNA damaging potential of nanoparticles. The results of this assay indicated that MMP2/LHRH Mn_3_O_4_ NPs and empty NLCs did not demonstrate signs of DNA damage and thus are safe for *in vivo* use.

Free anticancer drugs have large volumes of distribution and therefore may have adverse side effects on normal tissues, fast clearance, and reach insufficient concentrations at tumor sites. Delivery of anticancer drugs using nanoparticles has the realistic potential of overcoming these deficiencies. Lipid-based nanoparticles are composed of biocompatible materials and are ideal in incorporating lipohilic compounds. Unlike solid lipid nanoparticles, the inclusion of a liquid lipid in our formulation results in nanostructured lipid carriers. The liquid lipid (isopropyl myristate) fills the gaps in the crystal structure of the solid lipid (Precirol ATO 5). The drug molecule is solubilized in the liquid lipid portion of the formulation for NLCs as opposed residing in the gaps of the crystal structure of SLNs. Although NLCs have been shown to have higher entrapment of small drug molecules and less significant drug leakage, the liquid lipid portion appears to be the limiting factor for drug loading as the amount of drug entrapped is partially determined by its solubility in the liquid lipid. The current formulation was able to achieve 62.3% entrapment efficiency. Increasing the amount of drug in the pre-synthesis reaction did not increase loading. Therefore, the limiting factor may be the solubility of vemurafenib in isopropyl myristate.

The encapsulation of an anticancer agent within the matrix of a nanocarrier ensures that healthy tissues are not exposed to the substance. There was no release of vemurafenib from the liquid lipid filled matrix of the NLCs in neutral pH in PBS buffer for at least 72 h. Therefore, release of free drug would require nanoparticle digestion, which can occur in the lysosomal environment of cancer cells.

The cytotoxicity potential of the NLC entrapped vemurafenib formulation was assessed in Colo 829 human metastatic melanoma cell line. This cell line has the BRAF V600E, which is found in a large proportion of melanomas. Vemurafenib was developed as a molecularly targeted agent for this mutation. Because of its specificity to the mutation, vemurafenib should be effective in melanoma cells and not induce severe adverse side effects on normal cells. However, clinical use of the drug has shown the potential for the development of squamous cell carcinomas (58). Delivery within a NLC can increase accumulation in melanoma cells and spare healthy skin tissue.

MTT cytotoxicity assay in Colo 829 revealed that encapsulation of the drug into nanocarriers significantly increased its cytotoxicity when compared with free non-encapsulated vemurafenib. The comparison of these finding with other results of the present study shows the potential of the cancer-targeted Vem-NLCs formulations for decreasing systemic exposure to vemurafenib and increasing its cytotoxicity specifically in malignant melanoma cells.

Administration of MMP2/LHRH Mn_3_O_4_ NPs into a subcutaneous ovarian cancer model resulted in a significant increase in MR signal intensity. After 45 min, the signal had improved in average by 22.3%. The entire tumor mass showed an increase in signal demonstrating that the nanoparticles had preferentially accumulated in the tumors. This is important in the diagnosis of cancer because the more accurate determination of the size of the tumor can help in determining the next steps in therapy. High sensitivity of MRI combined the proposed imaging contrast NPs will help in early cancer diagnostics and detection of metastases. Also, imaging procedures can be used for monitoring therapy prognosis and decrease in tumor size. The preferential accumulation of cancer-targeted NPs in tumors was confirmed by monitoring the passage of the NPs from the administration site to the tumor xenograft with time. In addition to the subcutaneous model, Mn_3_O_4_ NPs were evaluated in orthotopic mouse models that resemble a similarity to cancer development in humans. The pre- and post-dose images demonstrate the power of the Mn_3_O_4_ NPs as MR imaging agents and showed a significant increase in the MR signal (Table II).

**Table II:** Increase in Magnetic Resonance Signal Intensity Post-Dose

	Post-Dose	15 min	30 min
Lung Cancer	No difference	+15%	No difference
Ovarian Primary Tumor and Malignant Ascites	Slight brightening	Delineation of tumor masses	Delineation of tumor masses
Primary and Metastatic Melanoma	+59.26%	+69.51%	+17.64%

Also, this experiment demonstrates that the particles are not simply deposited, but selectively accumulate in tumor tissue. In fact, the lungs without tumors demonstrated no signal even after injection of the contrast agent.

Although the intraperitoneal ovarian primary tumor with malignant ascites has some signal intensity without contrast administration, the post-dose image not only shows an increase in the signal but also demonstrated the delineation of the tumor shapes. Changing the targeting ligand from LHRH peptide to ab733 anti-melanoma monoclonal antibodies allows the particles to be adapted for use for detection of melanoma. The PEGylated Mn_3_O_4_ NPs provide an excellent foundation for further modification to meet required needs. The MMP2/ab733 Mn_3_O_4_ NPs showed increased signal in two sites of melanoma metastasis.

MRI revealed that the signal began to decrease 30 min after injection of NPs. The circulation time appears to be ample for tumor accumulation and short enough to prevent deposition in healthy tissues, which can limit potential adverse side effects. The circulation time is likely to be dependent on the size of the nanoparticles, but further studies will need to be conducted for the confirmation of such conclusion.

Compared to other studies using that also used manganese oxide based imaging agents for *in vivo* studies (2,3,22), the dose (Mn^2+^ concentration) in the current study was chosen to be at the substantially lower range. It is important to use the lowest effective dose to prevent toxicity especially for nanoparticles that will be administered repeatedly. The proposed Mn_3_O_4_ NPs have the potential to be effectively used for diagnosis of various cancers and monitoring therapy prognosis. The ability of the particles to clearly outline shape of tumor masses can be used to monitor decrease in tumor volume.

## CONCLUSION

An original design of cancer targeted responsive nanoparticle-based systems for magnetic resonance imaging and treatment of different cancers was proposed. Two types of nanoparticles were developed based on PEGylated water soluble Mn_3_O_4_ nanoparticles and nanostructured lipid carriers. The nanoparticles were modified with cancer-targeting moiety (LHRH peptide or anti-melanoma antibodies). The possibility of modification with other peptides (*e.g.* biotinylated MMP2 cleavable peptide) and encapsulation of anticancer drugs (*e.g.* vemurafenib) was also verified and tested. Synthesized nanoparticles were evaluated *in vitro* and *in vivo* using different models of cancer (transgenic mice developed melanoma, nude mice with orthotopic human lung and ovarian cancers, as well as subcutaneous xenografts of ovarian cancer). The results of the present investigation showed substantial advantages of the proposed nanoparticles as MRI contrasting agents. The usage of these nanoparticles significantly amplified a magnetic resonance signal specifically from cancer cells and improved the detection of primary tumors, precise determination of their shape, and visualization of metastases. Preferential accumulation of cancer targeted nanoparticles in cancer cells not only enhanced the sensitivity of MRI but also open a door for targeted treatment of various types of cancer.

## Abbreviations

BCA: Bicinchoninic acid
DMF: N,N-dimethylformamide
DMSO: Dimethyl sulfoxide
DOTAP: 1,2-dioleolyl-3-trimethylammonium-propane chloride salt
DSPE-PEG-COOH: 1,2-distearoyl-*sn*-glycero-3-phosphoethanolamine-N-[Carboxy(Polyethylene Glycol) 2000] ammonium salt
DSPE-PEG-MAL: 1,2-distearoyl-*sn*-glycero-3-phosphoethanolamine-N-[Maleimide(Polyethylene Glycol) 2000] ammonium salt
DSPE-PEG-NH_2_: 1,2-distearoyl-*sn*-glycero-3-phosphoethanolamine-N-[Amino(Polyethylene Glycol) 2000] ammonium salt
EDC: 1-ethyl-3-[3-dimethylaminopropyl]carbodiimide hydrochloride
EDTA: Ethylenediaminetetraacetic acid
HEPES: 4-(2-hydroxyethyl)-1-piperazineethanesulfonic acid
IACUC: Institutional Animal Care and Use Committee
LHRH: Luteinizing hormone releasing hormone
MMP2: Matrix metalloproteinase 2
MMP2/LHRH Mn_3_O_4_ NPs: Manganese oxide nanoparticles modified with MMP2 cleavable peptide and LHRH targeting peptide
MMP2/MAL Mn_3_O_4_ NPs: Manganese oxide nanoparticles modified with MMP2 cleavable peptide
MRI: Magnetic resonance imaging
NH_2_/MAL Mn_3_O_4_ NPs: Water soluble manganese oxide nanoparticles coated with DSPE-PEG-NH_2_ and DSPE-PEG-MAL
NHS: N-hydroxysuccimide
NP(s): Nanoparticle(s)
PBS: Phosphate buffered saline
SATA: S-acetylthioglycolic acid N-hydroxysuccinimide ester
SDS: Sodium dodecyl sulfate
Soy PC: Soy L-α-phosphatidylcholine
TMB: 3,3′,5,5′-tetramethylbenzidine
Vem: Vemurafenib
